# Cerebellar Infarction from a Vertebral Artery Dissection after Blunt Chest Injury: A Case Report

**DOI:** 10.5811/cpcem.1301

**Published:** 2023-10-20

**Authors:** Daniella Lamour, Joshua J. Solano, Jovana Rutherford, Scott M. Alter

**Affiliations:** * Florida Atlantic University, Charles E. Schmidt College of Medicine, Department of Emergency Medicine, Boca Raton, Florida; † Bethesda Hospital East, Department of Emergency Medicine, Boynton Beach, Florida; ‡ Delray Medical Center, Department of Emergency Medicine, Delray Beach, Florida; § St. George’s University School of Medicine, Grenada, West Indies

**Keywords:** *cerebrovascular injury*, *blunt chest trauma*, *vertebral artery dissection*, *cerebellar infarction*, *case report*

## Abstract

**Introduction:**

Traumatic vertebral artery dissections resulting in stroke are relatively rare occurrences, especially in the absence of classic physical examination findings.

**Case Report:**

We present the case of a 30-year-old male with chest pain following a car axle falling onto his chest while trying to change a tire. He was discharged from the emergency department after having a negative workup for thoracic injury. Six hours later, the patient returned with headache and was found to have a cerebellar stroke secondary to vertebral artery dissection. After hospitalization, the patient was discharged home without any neurological deficits.

**Conclusion:**

As they are usually asymptomatic, up to 80% of patients with blunt cerebrovascular injury will have delayed or missed diagnoses. Given the increased awareness of vascular injuries and their high morbidity, physicians should maintain a high index of suspicion for this diagnosis.

CPC-EM Capsule What do we already know about this clinical entity?

*Blunt cerebrovascular injury after trauma is rare. Patients with vertebral artery dissections are typically initially asymptomatic and have delayed presentations.*

What makes this presentation of disease reportable?

*The patient developed neurologic symptoms six hours after blunt chest injury and was found to have a vertebral artery dissection causing cerebellar infarction.*

What is the major learning point?

*Patients with vertebral artery dissections are usually asymptomatic. Up to 80% will be misdiagnosed or have a delayed diagnosis even with screening tools.*

How might this improve emergency medicine practice?

*Given the increased awareness of vascular injuries and the high incidence of morbidity, physicians should maintain a high index of suspicion for this diagnosis.*

## INTRODUCTION

While injuries are a leading cause of emergency department (ED) visits in the United States, blunt cerebrovascular-related trauma is rare.^1^ Blunt cerebrovascular injury (BCVI), a physical insult to the carotid or vertebral artery, occurs in less than 1% of blunt trauma patients and carries a high incidence of morbidity of 20–30%.^1^ Mechanisms leading to BCVI are exaggerated rotations of the neck with hyperextension and contralateral spin of the head.^2^ Furthermore, a direct blow to the carotid and vertebral arteries can lead to tearing of the tunica intima and media. A hematoma may develop intramurally, and neurological damage occurs from thromboembolism. Exposed endothelium stimulates platelet aggregation on arterial walls, causing luminal narrowing and occlusion, resulting in ischemia and brain infarction within days of injury.^3^ The high velocity of arterial pulses on irregular tears can cause arterial transection, possibly resulting in hemorrhagic stroke, ischemic stroke, pseudoaneurysm, and death. We report a case of a 30-year-old male who presented to the ED with chest pain after blunt chest trauma and returned six hours later with stroke-like symptoms secondary to vertebral artery dissection.

## CASE REPORT

A 30-year-old male with no significant medical history presented to a community ED with chest pain after a car axle fell onto his chest just before arrival. The patient was working beneath a Ford Mustang held up by a hydraulic jack. The jack broke and the axle fell on him. The patient momentarily lost consciousness. He was immediately removed from beneath the car. He was ambulatory and only complained of right-sided chest pain, rated 10/10, described as pressure-like without any radiation, worse with movement, and improved with rest. Family history was significant for a parent with cerebrovascular accident (CVA) at age 35 from a drug overdose.

**Image 1.**
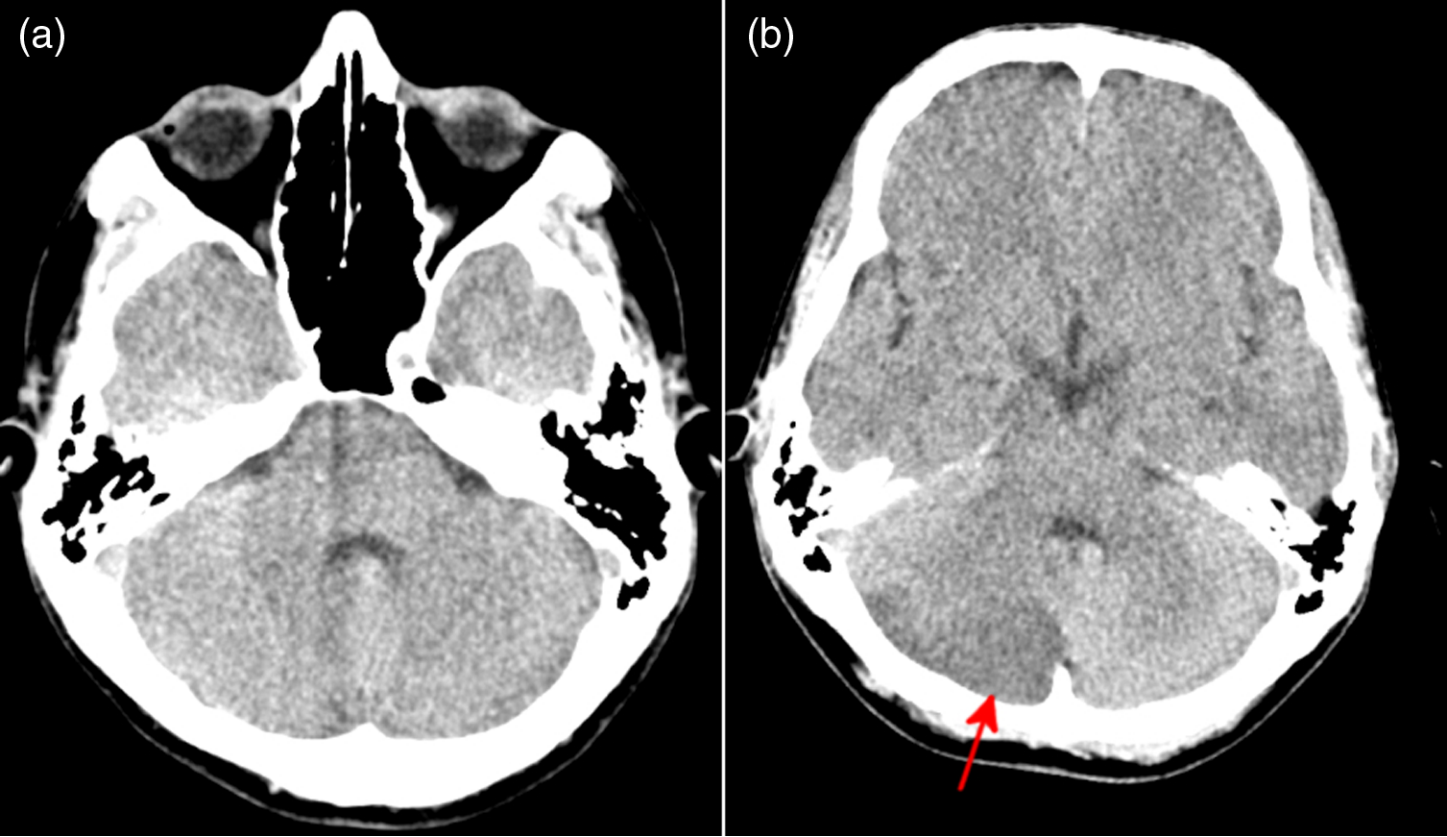
Computed tomography of the head on day of injury (a) just after initial injury showing no acute intracranial pathology, and (b) six hours after injury showing acute infarct involving the right cerebellar hemisphere (arrow).

Presenting vital signs were normal with pulse 82 beats per minute, respirations 18 breaths per minute, blood pressure 122/76 millimeters of mercury, temperature 98.3° Fahrenheit, and pulse oximetry 100% on room air. On physical examination, the patient had moderate chest wall tenderness along the right distal clavicle with surrounding erythema. He had painful right-shoulder range of motion with normal distal right arm motor, sensation, and pulses. The patient had a Glasgow Coma Scale (GCS) of 15 and no focal neurologic deficits. He was given morphine for pain. Due to the severe mechanism of injury, broad imaging was performed. Non-contrast computed tomography (CT) of the head and cervical spine were negative for acute injury (Image 1a), although the CT incidentally revealed a congenital segmentation anomaly involving the right lateral mass of the first (C1) and second (C2) cervical vertebrae, with incomplete C1 ring and dysplastic odontoid. Computed tomography of the chest, abdomen, and pelvis with intravenous (IV) contrast revealed a right distal clavicular fracture. Upon re-evaluation, the patient remained hemodynamically stable and neurologically intact. Pain improved to a 1/10. He was placed in a sling and was advised to follow up with an orthopedist and primary care physician within 48 hours. He was prescribed oxycodone-acetaminophen for pain and discharged.

**Image 2.**
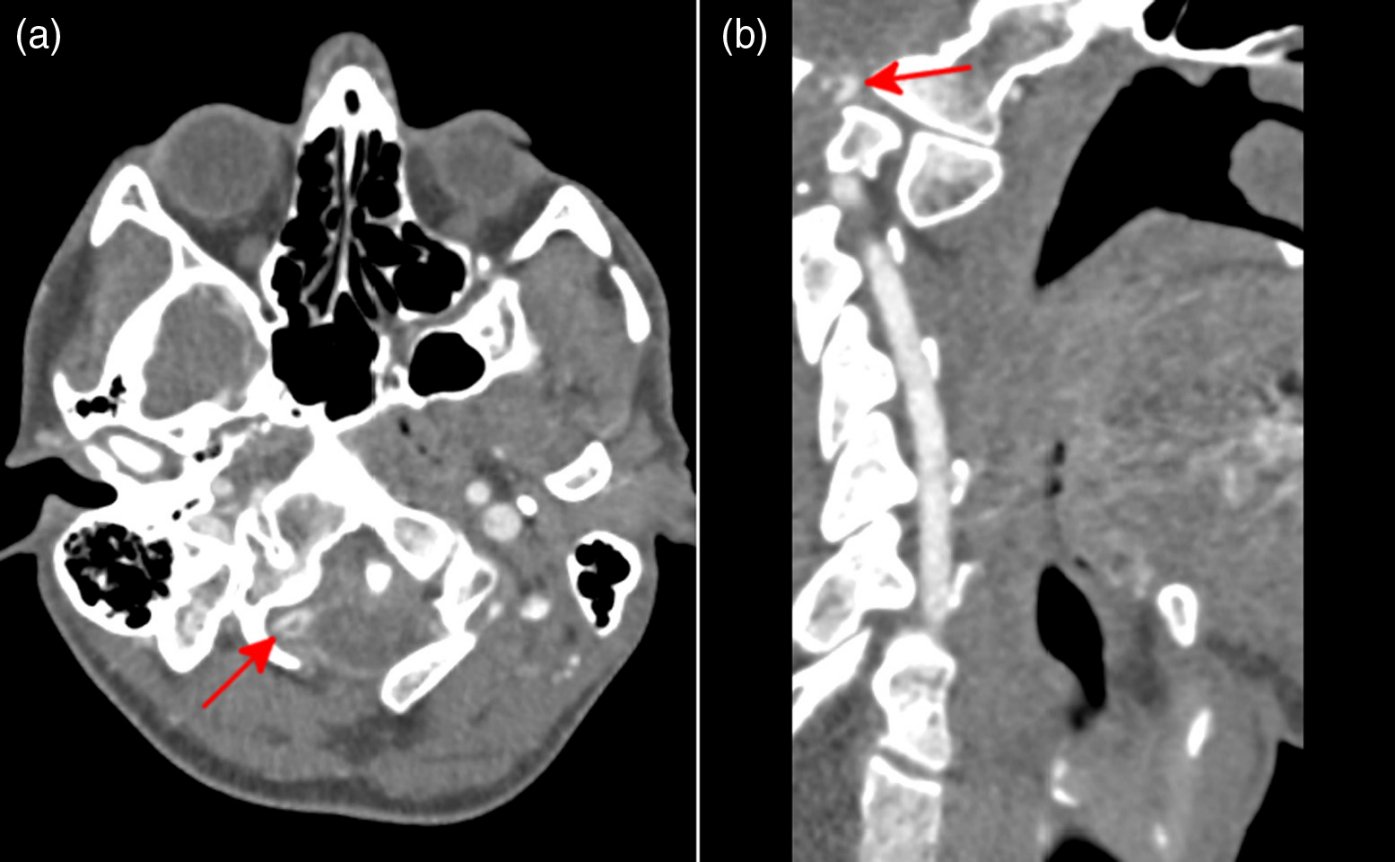
Computed tomography angiography of the head and neck showing short segment intimal dissection in the right vertebral artery just above the level of the foramen magnum (arrows) on (a) axial and (b) oblique views.

Six hours later, the patient returned to the ED after developing a gradual onset occipital headache, nausea, and vomiting. He took oxycodone-acetaminophen without relief of symptoms. On physical examination, the patient was ambulatory and neurologically intact with no focal neurological deficits. Cranial nerves II-XII were intact, there was 5/5 strength in bilateral upper and lower extremities, and there was normal sensation throughout. Repeat non-contrast head CT revealed an acute infarct involving the right cerebellar hemisphere, new from the prior study (Image 1b). A CT angiography (CTA) of the head and neck was then obtained, which revealed a short-segment intimal dissection in the right vertebral artery just above the level of the foramen magnum (Image 2). Again noted was the bony segmentation abnormality at C1-C2. Given the CVA was secondary to traumatic dissection, the patient was transferred to a trauma center.

Upon the patient’s arrival to the trauma center, he was in acute distress and actively vomiting. His neurologic examination was significant for a leftward nystagmus but was otherwise unremarkable. He was alert and oriented to person, place, time, and situation, cooperative, with normal and intact visual fields; extremity strength and sensation (although examination to right upper extremity was limited by a sling); cranial nerves II-XII (besides the nystagmus); gait; and deep tendon reflexes. A cerebral arteriogram was performed and revealed a traumatic dissection of the right vertebral artery third division at the skull base with resultant posterior inferior cerebellar artery (PICA) territory infarct with interval reperfusion and mild mass effect. Although the right vertebral artery dissection was not flow-limiting, it remained a risk factor for distal dissection. The neurointerventionalist felt there was a significant risk of hemorrhagic conversion in the PICA infarct, as he was re-perfused, and he was also at risk of needing a suboccipital decompression if the mass effect continued to increase. Therefore, the patient was initially treated with only aspirin 300 milligrams (mg) by rectum daily, later switched to 325 mg by mouth, rather than dual antiplatelet therapy or anticoagulation.

On day two of his hospital course, the patient developed mild hydrocephalus with mass effect on the fourth ventricle and ataxia on clinical examination. The patient was treated with 3% saline IV continuously at 100 milliliters per hour (mL/hr) for the first day, 50 mL/hr subsequently with a goal sodium level of 140–145 millimoles per liter, and later oral sodium chloride tabs 1 gram orally three times per day. He was also treated with dexamethasone 4 mg IV every six hours for the first two days and then switched to an oral taper. Symptoms waxed and waned, but after one week of treatment, symptoms improved and repeat head CT showed decreased mass effect with diminished hydrocephalus. The patient was discharged after a 10-day hospitalization with a completely normal neurological exam. In follow-up two weeks later, repeat head CT revealed ongoing aging of the right cerebellar infarct, which had decreased in size, showing primarily chronic-appearing features, near complete hydrocephalus resolution, and no intracranial hemorrhage midline shift or mass effect.

## DISCUSSION

Blunt cerebrovascular injury after blunt trauma is rare, historically reported to be less than 1%, but now upward to about 2% given an increase in awareness.^1^ Of all strokes, only 2.6 per 100,000 are caused by vertebral artery dissection.^2^ Our patient initially presented with blunt chest injury, with no symptoms concerning for vertebral artery dissection. This presentation is common as patients with vertebral artery dissections are typically asymptomatic immediately after the injury. One study noted an 18-hour delay between traumatic dissection-causing injury and symptom onset.^4^ Often, dissections are not diagnosed until patients present with neurologic deficits secondary to strokes or transient ischemic attacks, as with our patient. Our patient likely had the vertebral artery dissection upon initial presentation, although asymptomatic at the time, perhaps caused by rapid extension and rotational movement of his neck as the vehicle fell on him.

Vertebral artery dissections occur when a tear in the intimal layer of the vessel exposes endothelium, which stimulates platelet aggregation and formation of a thrombus to initiate vessel repair.^5^ Stoke symptoms subsequently occur due to vessel occlusion either at the dissection site by the intimal flap or thrombus or, more distally by thrombus embolization into the cerebral circulation.^3^ These differing mechanisms explain why symptom presentation onset vary. In our patient, the dissection likely occurred immediately after the accident but went undetected because vessel occlusion had not yet occurred. The vertebral artery supplies the spinal cord, brainstem, cerebellum, and posterior brain. Dissections most commonly occur superior to the C2 vertebra where the artery is mobile and not anchored as it ascends into the foramen magnum,^5^ ^,^ ^6^ which was the location of our patient’s dissection. His variant C1-C2 bony anatomy may have predisposed him to the injury, with the vertebrae providing less protection and allowing for more mobility of the vessel.

To help identify who requires a CTA to evaluate for BCVI, the Denver criteria were developed and include the following: arterial hemorrhage; cervical bruit; expanding hematoma; focal neurological deficit; and stroke on CT.^7^ Risk factors include high-energy mechanism with cervical spine fracture, LeForte II or III fracture, basilar skull fracture with carotid canal involvement, diffuse axonal injury with GCS less than six, or near-hanging with anoxic brain injury.^7^ ^,^ ^8^ Another study revealed cervical spine, facial, and basilar skull fractures were the strongest predictors of BCVI.^9^ Our patient presented with a stroke on secondary CT, prompting further imaging, leading to diagnosis. He was then treated with anti-platelet therapy to decrease the risk of hemorrhagic conversion. Treatment of vertebral artery dissections consists of thrombolytic therapy if presenting within 4.5 hours, anti-platelet therapy, or anticoagulation. Endovascular or open operative repair are usually reserved for high-grade lesions.^8^ Despite ongoing cerebellar infarct at two-week follow-up, our patient had a full neurological recovery.

## CONCLUSION

Cerebellar infarctions secondary to vertebral artery dissections are rare. As they are usually asymptomatic, up to 80% of patients will be misdiagnosed or have a delayed diagnosis even with screening tools. Our patient, with variant C1-C2 anatomy and blunt upper chest injury, had risk factors for vertebral artery dissection. Given the increased awareness of vascular injuries and their high incidence of morbidity, physicians should maintain a high index of suspicion for this diagnosis.

## References

[1] Hundersmarck D Slooff WM Homans JF et al. Blunt cerebrovascular injury: incidence and long-term follow-up. Eur J Trauma Emerg Surg. 2021;47(1):161–70. 31197394 10.1007/s00068-019-01171-9 PMC7851103

[2] Yakupoğlu A Korkmazer B Arslan S et al. Vertebral artery dissections: a retrospective data analysis of a single center. Cerrahpaşa Med J. 2022;46(3):183–8.

[3] Nagpal P Policeni BA Bathla G et al. Blunt cerebrovascular injuries: advances in screening, imaging, and management trends. AJNR Am J Neuroradiol. 2017;39(3):406–14. 29025722 10.3174/ajnr.A5412 PMC7655313

[4] Biffl WL Moore EE Offner PJ et al. Optimizing screening for blunt cerebrovascular injuries. Am J Surg. 1999;178(6):517–22. 10670864 10.1016/s0002-9610(99)00245-7

[5] Testai FD and Gorelick PB. An unusual cause of vertebral artery dissection: esophagogastroduodenoscopy. Stroke Res Treat. 2010;2010:915484. 20847949 10.4061/2010/915484 PMC2934772

[6] Gailloud P. The segmentation of the vertebral artery: an ambiguous anatomical concept. Interv Neuroradiol. 2022;28(6):765–72. 34866439 10.1177/15910199211063275 PMC9706265

[7] Desouza RM Crocker MJ Haliasos N et al. A. Blunt traumatic vertebral artery injury: a clinical review. Eur Spine J. 2011;20(9):1405–16. 21674212 10.1007/s00586-011-1862-y PMC3175894

[8] Shafafy R Suresh S Afolayan JO et al. Blunt vertebral vascular injury in trauma patients: ATLS recommendations and review of current evidence. J Spine Surg. 2017;3(2):217–25. 28744503 10.21037/jss.2017.05.10 PMC5506306

[9] Leichtle SW Banerjee D Schrader R et al. Blunt cerebrovascular injury: the case for universal screening. J Trauma Acute Care Surg. 2020;89(5):880–6. 32520898 10.1097/TA.0000000000002824

